# Baleen whales host a unique gut microbiome with similarities to both carnivores and herbivores

**DOI:** 10.1038/ncomms9285

**Published:** 2015-09-22

**Authors:** Jon G. Sanders, Annabel C. Beichman, Joe Roman, Jarrod J. Scott, David Emerson, James J. McCarthy, Peter R. Girguis

**Affiliations:** 1 Department of Organismic and Evolutionary Biology, Harvard University, 16 Divinity Avenue room 3085, Cambridge, Massachusetts 02138, USA; 2 Department of Ecology and Evolutionary Biology, University of California, Los Angeles, 610 Charles Young Drive South, Los Angeles, California 90095, USA; 3 Gund Institute for Ecological Economics, University of Vermont, 617 Main Street, Burlington, Vermont 05405, USA; 4 Bigelow Laboratory for Ocean Sciences, 60 Bigelow Drive, East Boothbay, Maine 04544, USA

## Abstract

Mammals host gut microbiomes of immense physiological consequence, but the determinants of diversity in these communities remain poorly understood. Diet appears to be the dominant factor, but host phylogeny also seems to be an important, if unpredictable, correlate. Here we show that baleen whales, which prey on animals (fish and crustaceans), harbor unique gut microbiomes with surprising parallels in functional capacity and higher level taxonomy to those of terrestrial herbivores. These similarities likely reflect a shared role for fermentative metabolisms despite a shift in primary carbon sources from plant-derived to animal-derived polysaccharides, such as chitin. In contrast, protein catabolism and essential amino acid synthesis pathways in baleen whale microbiomes more closely resemble those of terrestrial carnivores. Our results demonstrate that functional attributes of the microbiome can vary independently even given an animal-derived diet, illustrating how diet and evolutionary history combine to shape microbial diversity in the mammalian gut.

## Abstract (web summary)

Diet is a major factor determining the composition of gut microbiota in mammals, while host evolutionary history seems to play an unclear role. Here, Sanders *et al*. show that baleen whales, which prey on animals, harbour a unique gut microbiome with similarities to those of terrestrial herbivores.

## Graphical abstract

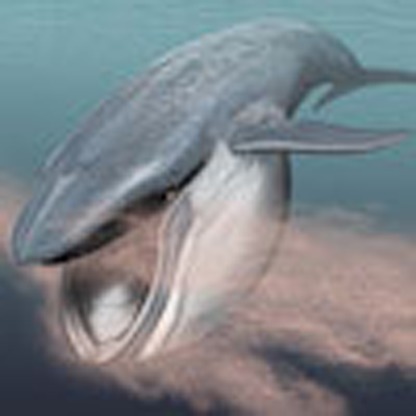

Mammals host gut microbiomes that are immensely important to the health, and likely fitness, of the host^1^,^2^. While the composition of these microbial communities is largely determined by host diet^3^,^4^, a substantial amount of variation is correlated with phylogeny: some mammals with diets atypical of their clade, such as the herbivorous panda bear, host communities that are taxonomically more similar to their close relatives than to other mammals with similar diets^3^,^5^. The determinants of this ‘phylogenetic inertia' are not yet well-understood, in part because of a paucity of data from mammals, like pandas, whose diets differ from those of their evolutionary ancestors.

Cetaceans (whales and dolphins) evolved from herbivorous terrestrial artiodactyls related to cows and hippopotamuses^6^, but feed exclusively on animals. Baleen whales are filter feeders, primarily consuming small schooling fish and crustaceans with chitin-rich exoskeletons (chitin is an abundant structural polysaccharide commonly found in marine habitats). On land, the closest extant relatives of whales are herbivores whose diets are rich in cellulose, another extremely abundant structural polysaccharide^7^. Despite these differences in diet, both whales and their ruminant relatives have multichambered foreguts^6^,^8^. Together, these factors make the whale microbiome an important point of reference for understanding the roles of diet and phylogeny in structuring mammalian gut communities. Yet, to date, the diversity and functional potential of these communities remain unknown.

Here we set out to better understand the diversity and functional potential of the baleen whale microbiome. We characterize the community composition of cetacean gut microbiomes via 16S ribosomal DNA and shotgun metagenomic sequencing of faecal samples. We compare data from several baleen and toothed whales to similar data from terrestrial mammals from this and previous studies. We find that baleen whales host a microbiome distinct from those of terrestrial mammals, but with functional similarities to both terrestrial carnivores and herbivores—including genes likely to be involved in fermenting polysaccharides from the exoskeletons of their prey.

## Results and discussion

### 16S community composition

**Figure 1:**
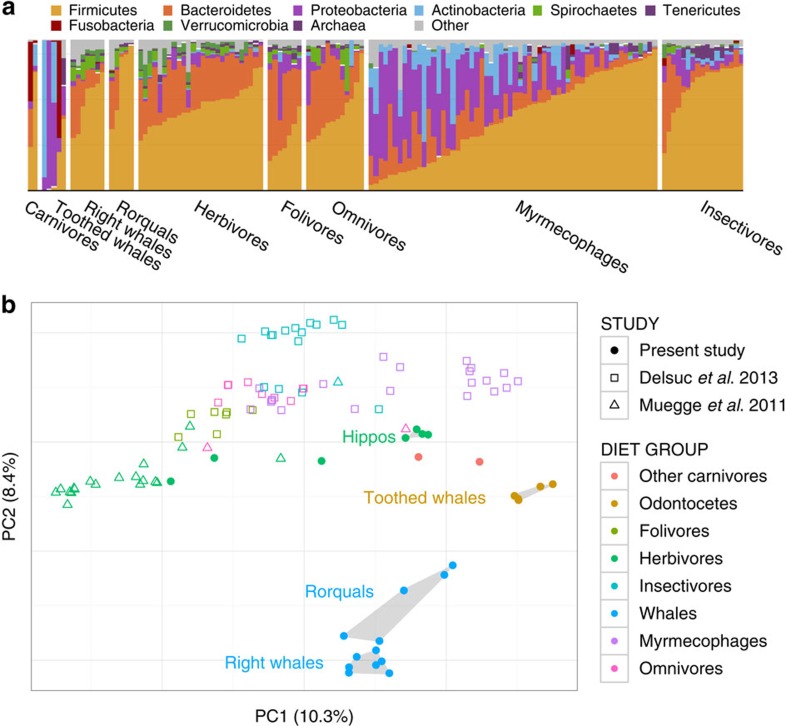
Baleen whales host a distinct microbiota. (**a**) Taxonomic composition of 16S rRNA amplicon sequences in cetaceans and terrestrial mammals. (**b**) PCoA ordination of unweighted UniFrac distances among mammalian gut microbiota. Note that despite similarity at higher taxonomic levels between the microbiota of baleen whales and those of terrestrial herbivores (**a**), baleen whales' microbiota are quite distinct from those of all sampled terrestrial mammal microbiota when considering individual OTUs (**b**) (i.e. reads with 97% similarity).

We found that, like most terrestrial mammals, baleen whale microbiota were dominated in large part by bacteria in the phyla Bacteroidetes and Firmicutes (Fig. 1a), though with a consistently higher proportion of reads assigned to the phyla Firmicutes and Spirochaetes and the classes Bacteroidia and Clostridia (see Supplementary Table 1). Baleen whale samples had either very few or no reads assigned to Proteobacteria, the Bacilli, members of the genus *Coprobacillacus* within the Erysipelotrichaceae, or members of the genera *Blautia* and *Coprococcus* within the Lachnospiraceae, all of which were comparatively common among terrestrial mammals (Supplementary Data 1). Some of these lineages were especially depleted in the right whales compared with the other baleen whales (humpback and sei whales; Supplementary Data 2), which also grouped closer to terrestrial mammals on a PCoA ordination of UniFrac distances (Fig. 1b). These two groups of whales consume substantially different diets, with right whales especially dependent on comparatively chitin-rich calanoid copepods, and the other whales also consuming fish, krill and copepods; these differences are discussed further below.

Several lineages that were comparatively abundant in baleen whale samples were also relatively enriched in terrestrial herbivores, including the clade 5 Verrucomicrobia, the phylum Lentisphaerae, the clade RF3 Tenericutes and the genus *Treponema* in the Spirochaetes. Notably, these groups were abundant in both ungulate and non-ungulate terrestrial herbivores, suggesting that these bacteria may play a similar role in each herbivorous host lineage—and that their shared presence in whales may be due as much to a functionally convergent role in the community as to phylogenetic conservation from the whales, ancestor. The toothed whale microbiota were highly variable in composition and are discussed elsewhere (see Supplementary Information).

Although the broad higher-taxonomic composition of the baleen whale microbiota was similar to those of terrestrial mammals, it was strikingly different at the level of 97% similarity operational taxonomic units (97% OTUs, Fig. 1b). Of 397 OTUs with an average abundance of at least 10 reads per whale, 87.9% were significantly different in abundance when compared with terrestrial mammals (Supplementary Data 3). As with terrestrial carnivores, our whale samples had comparatively low diversity, with toothed whales especially depauperate (Supplementary Fig. 1 and see Supplementary Information). Targeted exploration of the most abundant baleen whale OTUs suggests that the differentiation from observed terrestrial OTUs may have derived from a long separation from terrestrial animal sources rather than acquisition from marine animal microbiota or freeliving marine microbes: the top BLAST hits to these sequences are primarily to terrestrial animal gut bacteria (Supplementary Data 4). We also found little evidence of sequences derived from food or the environment in baleen whale samples: OTUs that had a 97% identity to a library of bacterial 16S sequences from North Atlantic copepods, the preferred food of right whales, accounted for 6 or fewer of the 10,000 rarified sequences in each of the baleen whale samples (Supplementary Fig. 2).

### Metagenome functional capacity

Given the whales' animal-based diet, the similarity in higher taxon distribution between the microbiota of baleen whales and those of terrestrial herbivores was striking—especially given the large differences in OTU composition. To help establish whether this higher order compositional similarity has functional underpinnings, we sequenced and analysed the predicted coding content of shotgun metagenomes for eight baleen whales. Our results showed that the whale microbiome echoes the carnivore microbiome for genes related to protein digestion and biosynthesis, but has similarity to herbivore microbiomes with respect to genes involved in carbon and energy metabolism—likely reflecting the shared relevance of polysaccharide fermentation to their gut microbial community.

**Figure 2:**
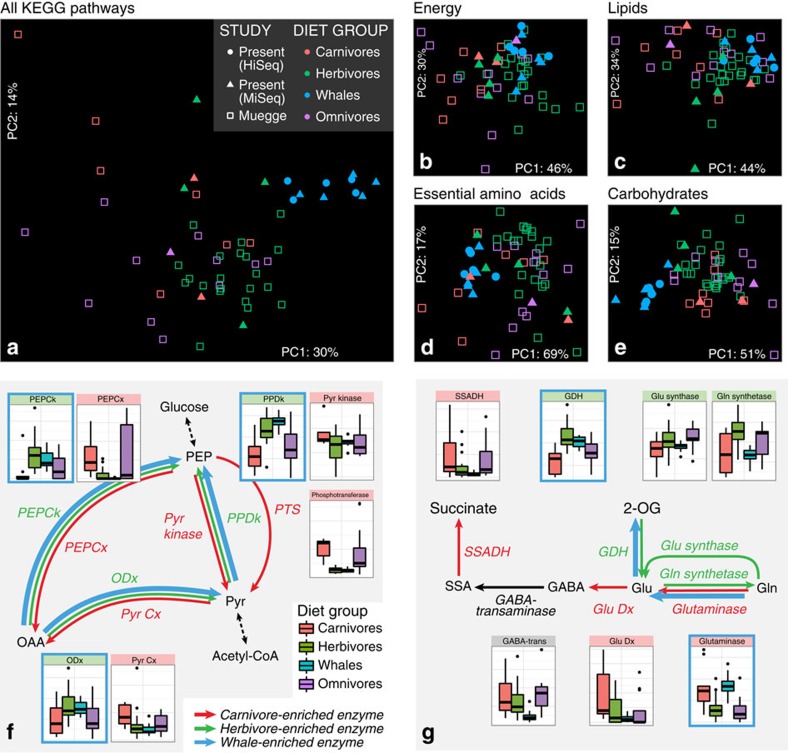
The functional compositions of baleen whale microbiomes show similarity to those of both terrestrial herbivores and carnivores. Principal components analysis ordinations of predicted metagenomic functional potential show baleen whales microbiomes are distinct from those of terrestrial mammals when considering all pathways (**a**) or pathways involved in carbohydrate metabolism (**e**). Pathways involved in energy (**b**) and lipid (**c**) metabolism are more similar to those of terrestrial herbivores, and pathways involved in the synthesis of essential amino acids (**d**) are more similar to those of terrestrial carnivores. Sub-pathways previously shown to separate herbivore and carnivore metagenomes show a similar split, with baleen whale metagenomes showing a pattern of enrichment similar to herbivores in central pyruvate metabolism (**f**) and to carnivores in glutamate metabolism (**g**). Distributions of normalized abundances are shown as box plots for each gene, coloured according to dietary category (green=herbivore, red=carnivore, purple=omnivore, blue=whale). Box plot whiskers extend to 1.5 times the interquartile range. Genes relatively enriched in terrestrial herbivores and carnivores in the analysis of data from ref. 4 have headers coloured green and red, respectively. Those enriched in whale microbiomes are outlined in blue, and the proposed direction of metabolite flux for each dietary category given as coloured arrows between metabolites.

Though 16S diversity may poorly predict specific subsets of functional potential^9^, investigators have observed that the general functional profile of a microbiome can be reasonably represented by the patterns observed in 16S-based community profiles^4^,^10^. The overall functional composition in our data set conformed to these expectations (Fig. 2a). In ordinations of predicted KEGG pathway abundances (Fig. 2a) and gene abundances (Supplementary Fig. 3a), the baleen whales formed a cluster distinct from terrestrial mammals, with the humpback and sei whales set slightly closer to terrestrial mammals, on average, than were the right whales—perhaps reflecting functional consequences of the aforementioned differences in diet between these groups of whales.

Although baleen whales grouped independently from terrestrial mammals when considering the entire set of KEGG genes or pathways, they tended to cluster with different terrestrial dietary groups at lower levels of the KEGG hierarchy. Baleen whale microbiomes were relatively similar to those of terrestrial carnivores in the abundance of pathways involved in essential amino acid metabolism and biosynthesis (multivariate effect size *η*^2^=0.15 versus carnivores and 0.71 versus herbivores, Fig. 2d), likely reflecting high quantities of protein in both diets. Both the baleen whale and terrestrial carnivore microbiomes were relatively depleted in genes encoding enzymes catalysing the final steps in biosynthesis of most essential amino acids (Supplementary Fig. 4). Baleen whale microbiomes were also enriched in genes catalysing the degradation of glutamine and glutamate, and depleted in genes catalysing the synthetic reactions (Fig. 2g). Glutamate metabolism is one of the most diagnostic pathways in distinguishing between microbiomes exposed to predominantly animal- and plant-based diets, both in humans^11^ and in mammals generally^4^. Together, these genes suggest a predominantly catabolic direction of protein metabolism, reflecting the whales' animal-based diet.

In contrast to this largely ‘carnivorous' profile for genes involved in amino acid metabolism, baleen whale microbiomes were considerably more similar to those of terrestrial herbivores when considering pathways in the KEGG categories of energy metabolism (*η*^2^_carnivore_=0.69, *η*^2^_herbivore_=0.24, Fig. 2b) and lipid metabolism (*η*^2^_carnivore_=0.56, *η*^2^_herbivore_=0.31, Fig. 2c). Like glutamate metabolism, pyruvate metabolism has been shown to be a key indicator in differentiating herbivorous and carnivorous microbiomes^4^,^11^. Similar to terrestrial herbivores, baleen whales were enriched in genes withdrawing metabolic intermediates from the tricarboxylic acid cycle, and depleted in genes catalysing the reverse reactions (Fig. 2f).

### Evidence for fermentative metabolisms

These similarities in carbon metabolism between the microbiomes of animal-eating baleen whales and plant-eating terrestrial herbivores may be explained by a shared reliance on fermentation. Like their herbivorous artiodactylate ancestors, whales possess a multichambered stomach^6^,^8^. Baleen whales also possess a blind-end caecum between the ileum and colon, which is not present in the toothed whales^8^. Given these morphological similarities to terrestrial herbivores, questions about the mechanisms and importance of chitin digestion in baleen whales have been considered for decades^12^,^13^. While one study found short chain fatty acids (SCFAs, the presumed end products of microbial fermentation) in grey and bowhead whale forestomachs that were comparable in concentration and composition to those in the forestomachs of terrestrial ruminants^12^, the importance of fermentation for whales has been contested, with others arguing that the rates of SCFA production, based on *in vitro* measurements, would only account for a small fraction of the animal's daily energy budget^13^. Similarly, to date there has been little evidence of methanogenesis in whale guts, a microbially mediated process commonly associated with fermentation in terrestrial animals^12^,^13^,^14^.

Although it is impossible to draw strong inferences about metabolite flux solely from potential metagenomic capacity, our data reveal that baleen whale microbiomes are markedly similar to those of terrestrial herbivores in the abundance profiles of genes associated with fermentation. This finding is consistent with a major role for fermentation in the whale gut microbiome for at least four reasons. First, baleen whale microbiomes were considerably more similar to those from terrestrial herbivores than to those from terrestrial carnivores in the abundance of genes related to the metabolism of pyruvate, a major fermentation intermediate. Second, we found that both whale and herbivore microbiomes were enriched in enzymes catalysing the production and utilization of SCFAs (Supplementary Fig. 5), consistent with measurements of high concentrations of SCFAs in baleen whale foreguts. Third, we found that enzymes primarily associated with the Wood–Ljungdahl pathway, a microbial pathway that consumes excess hydrogen produced during fermentation, were enriched in both whale and herbivore microbiomes relative to those of carnivores (Supplementary Fig. 6a). Finally, enzymes associated with hydrogenotrophic methanogenesis (for example, heterodisulfide reductase and methyl-coenzyme M reductase) were proportionally more abundant in both baleen whales and herbivores, suggesting that these whales' microbiomes do indeed retain the capacity for methanogenesis (Supplementary Fig. 6b). The baleen whale microbiota were devoid of 16S amplicons allied to the Methanobacteria, the group of Archaea classically associated with methanogenesis in mammal guts, and which were recovered at moderate abundance in many of the analysed terrestrial microbiota. However, both the baleen whales and terrestrial herbivores had a substantial number of reads allied to the recently described Euryarchaeal order Methanomassiliicoccales^15^,^16^. These obligate hydrogen-consuming methanogenic methylotrophs have to date been found exclusively in gut habitats, including humans, termites and cattle, and have recently been shown to be important methane producers^17^. Notably, some of these organisms can utilize methylated amines, which are abundant solutes in marine zooplankton^18^, as a substrate^17^.

Baleen whale microbiomes grouped independently from the terrestrial mammals only in the category of carbohydrate metabolism, likely reflecting fundamental differences in the source of carbohydrates (Fig. 2e). Fermentation in terrestrial herbivores is fed largely by plant-derived cellulose and hemicellulose, while the predominant carbohydrate source in many baleen whales is likely to be the chitinous exoskeleton of invertebrate prey such as krill, where chitin can account for as much as 10% of total calories^19^. Differences in these feedstocks are reflected in the monosaccharide kinase profiles of these groups. Terrestrial herbivore microbiomes are enriched in kinases for galactose, rhamnose and xylulose, three major constituents of hemicellulose that are absent in chitin (Supplementary Fig. 7). By contrast, whale, terrestrial herbivore and terrestrial carnivore microbiomes had similar representations of gluco- and fructokinases.

### Genes encoding carbohydrate-active enzymes

**Figure 3:**
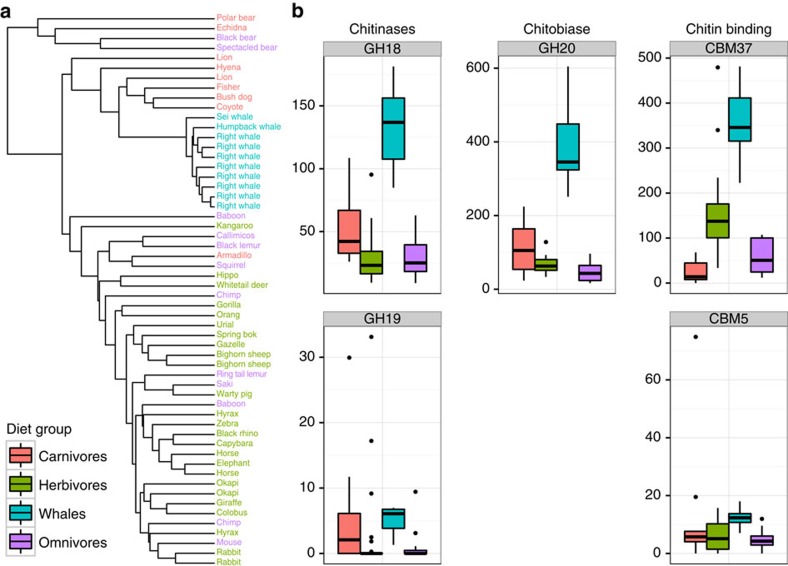
The composition of CAZymes in baleen whale microbiomes is distinct and enriched in genes predicted to have activity on chitin. (**a**) UPGMA-clustering dendrogram of CAZyme abundances. Dietary compositions are indicated by tip label colour. (**b**) Box plots showing distribution in normalized abundance of the five most abundant chitin-related CAZymes significantly enriched in baleen whale relative to terrestrial microbiomes. Note that CBM5 and CBM37 are binding domains rather than enzymes and CBM37 has activity against a broad spectrum of polysaccharides in addition to chitin^20^.

To further examine carbohydrate utilization in the baleen whale gut microbiome, we used a combination of sequence similarity and hidden Markov model searches to generate profiles of carbohydrate-active enzymes (CAZymes) for whale and terrestrial microbiomes. Baleen whales hosted a unique complement of these genes, consistent with the hypothesis that fermentation of animal carbohydrates is especially important in this community. UPGMA clustering of CAZy abundance profiles showed baleen whales grouping separately from terrestrial mammals, but nested within the carnivores rather than herbivores—reflecting the shared importance of animal polysaccharides in whale and terrestrial carnivore diets, despite the whales' similarities to herbivores in downstream fermentation pathways (Fig. 3).

Among the 179 CAZy families represented in the total data set, 36 were significantly more abundant in baleen whale metagenomes than in those of terrestrial mammals (Supplementary Data 5). These families are predominantly associated with the degradation of animal-derived polysaccharides. Nine of the significantly enriched families are described to have activity on chitin and associated compounds, including enzymes associated with chitin binding, hydrolysis of long-chain chitin polymers (chitinases) and hydrolysis of chitin oligomers (chitobiases). Genes with potential for activity on chitin were also especially abundant as an overall fraction of carbohydrate-active genes. The chitobiase GH20 was on average the third most abundant CAZyme in the baleen whale metagenomes, and was the most abundant of the enriched CAZymes (Fig. 3). Nearly as abundant was the carbohydrate-binding module CBM37, which has been shown to bind to a number of substrates, including chitin^20^. To date, this module has only been described from the bacterial genus *Ruminococcus*, where it has been proposed to function in the localization of glycanases to the bacterial cell wall for attachment to and degradation of extracellular crystalline polysaccharides^21^. Together with the chitinase GH18, these CAZymes comprised three of the six most abundant families enriched in baleen whales.

The distribution of CAZy families in the whale gut likely reflects one of the major axes of variation in both taxonomy and function relative to other currently known mammalian microbiomes. Profiles of CAZy families have been found to be strong predictors of diet in mammalian and human microbiomes^22^, and different microbial lineages seem to be consistently associated with particular carbohydrate categories. Bacterial isolates in the genus *Bacteroides*, for example, have thus far been found to encode especially large numbers of animal-carbohydrate active enzymes^22^,^23^. Consistent with this pattern, our baleen whale samples were relatively enriched in 16S sequences assigned to the genus *Bacteroides* (Supplementary Data 1). The profile of CAZyme diversity within bacterial lineages appears to be quite labile, however^9^, and more targeted work will be necessary to identify the functional roles of specific bacteria within the whale gut.

## Conclusions

The parallels in higher taxa composition between the microbiota of terrestrial herbivores and the baleen whales in this study help to clarify the complex interplay between the dietary and physiological determinants of the mammalian gut microbiome. As in previous surveys of mammalian gut microbiota, our data show correlations with both diet and host phylogeny^3^,^4^, depending on which dimensions of the data are being considered. However, in baleen whales, the correlation between host phylogeny and microbiome composition may reflect constraints imposed by the physical structure of the gastrointestinal tract itself, with the multichambered artiodactyl foregut serving as a preadapted fermentation chamber. Metagenomic and metatranscriptomic profiling along the length of the baleen whale gut will help to clarify the relationship between gut morphology and microbial function in whales: while faecal samples are useful for comparisons across taxa, and retain a signature of fermentation in foregut-fermenting herbivores^4^, they integrate microbial DNA from along the gut, and can differ significantly from samples taken from proximal gut compartments^24^. Under the model proposed here, it is this shared complex forestomach that sets the stage for the degradation of chitin—a polysaccharide with some structural similarities to cellulose, but requiring an entirely different set of CAZymes for digestion—to drive a fermentative profile for overall carbon metabolism in the microbiome that is in many respects similar to that observed in the whales' terrestrial herbivore relatives.

A multichambered foregut may also help to explain the lack of affinity we observed between the microbiota of baleen whales and those of terrestrial insectivores and myrmecophages (obligate ant- and termite-eaters). These carnivorous mammals also eat chitin-rich arthropod-based diets, but unlike whales they have relatively simple guts. As has been described in pandas, whose simple guts do not appear to sustain large quantities of typically cellulolytic bacteria^25^, the simple guts of these terrestrial insectivores may not provide access to more complex polysaccharides via fermentation. Although we only had access to metagenomic data for two terrestrial mammals likely to consume substantial quantities of arthropods (an armadillo and an echidna), the CAZy profiles of these specimens did not cluster closely to those of the baleen whales (Fig. 3), nor were they especially enriched in chitin-related genes relative to terrestrial carnivores. Future surveys of the functional potential of insectivorous mammalian microbiomes should help to further clarify the role of host gut physiology in modulating the microbiome's response to dietary input^26^.

The multichambered foregut may also have played an important role in the evolution of the baleen whales by facilitating the maintenance of a microbial community capable of extracting relatively recalcitrant nutrients from zooplankton. Although empirical estimates of the microbial contribution to nutrition in whales vary^13^, chitin alone may comprise upto 7% of the total dry weight and 7–10% of total caloric content of krill and copepods^19^,^27^,^28^. Baleen is a relatively recent innovation, and the whales' most recent common ancestor was likely piscivorous^6^. Consequently, this most recent common ancestor may have had a gut microbiome more similar to modern-day dolphins and seals^29^. More extensive sampling of toothed whales (see Supplementary Discussion) will help to further constrain the relative roles of diet, environment and phylogeny in structuring the diversity of mammalian gut microbiomes.

Finally, our results highlight the potential impact of the multichambered artiodactyl gut on global marine biogeochemistry. Using many of the same fermentative pathways and higher microbial taxa that their terrestrial relatives employ to utilize cellulose, the most abundant biopolymer on land, whales evolved a process to utilize chitin, the most abundant biopolymer in the sea^7^. Given this abundance, and its consequent role as a major marine reservoir of both carbon and nitrogen, broad questions about the distribution of chitin-degrading microbes in the sea have been pursued for over 75 years^30^. The digestive capacity of baleen whales has consequences for elemental flux throughout the ocean, including enhanced benthic-pelagic coupling; increased marine productivity as a result of the near-surface release of nutrient-rich faecal plumes; the transfer of nutrients to areas of low productivity during migration; and organic enrichment of the deep sea via whale carcasses^31^. Such processes may owe as much to the microbes in the belly of the whale as to the whales themselves.

## Methods

### Sample collection

Baleen whale faecal samples for this project were collected primarily in August 2011 from whales feeding in the Bay of Fundy, located between New Brunswick and Nova Scotia, Canada. Fisheries and Oceans Canada granted permits to J.R. and J.J.M. to collect whale faeces in the Bay of Fundy (license #325842). Collection methods have been described previously^32^. Briefly, whales were located and identified visually, and associated faecal samples were collected as quickly as possible post defecation using either a mesh dip net or a cod-end plankton net with a 150-μm mesh size. Approximately 10 ml of each faecal sample was transferred to a sterile plastic tube and placed immediately on ice. These samples were then frozen at −20 °C on return to shore (see Supplementary Table 1 for full collections information).

Faecal samples from these whales were complemented by a variety of faecal specimens from wild and captive marine mammals. Faeces from Pacific Humpback Whales (*Megaptera novaeangliae*) from Sitka Sound and the Seymour Canal in Southeast Alaska were provided by Prof Jan Straley, collected under NOAA permits 474-1700-02 and 14122. One specimen, from the Atlantic white-sided dolphin (*Lagenorhynchus acutus*), was collected from the colon during necropsy of a beached wild individual (under Fisheries of Canada license #325842). Two samples each were collected from captive bottlenose dolphins (*Tursiops truncatus*) and Beluga whales (*Delphinapterus leucas*) from the Long Marine Lab at the University of California at Santa Cruz and Mystic Aquarium in Mystic, Connecticut, respectively. Captive toothed whale samples were collected immediately following defecation by target individuals and frozen at −20 °C.

To enhance the number of terrestrial mammal samples for comparison, and to facilitate comparison of our data against previously published data for terrestrial mammals, additional samples were collected from terrestrial and aquatic mammals. Four faecal samples from wild hippopotamus (*Hippopotamus amphibius*) were provided by Dr Amanda Subalusky (Yale University). These samples were collected from fresh dung pats and preserved in the field using RNALater (Ambion Inc) at room temperature, then transferred to 4 °C on return to the US, and kept at 4 °C until DNA extraction. Fresh scat from wild carnivores and herbivores was collected by members of the volunteer wildlife tracking group Keeping Track Massachusetts (keepingtrack.org). Additional specimens from captive herbivores were collected by the authors (for detailed collections information, see Extended Data Table 1). All of these terrestrial samples were frozen at −20 °C as soon as possible after collection, and remained frozen until DNA extraction.

### DNA extraction and sequencing

DNA was extracted from samples using MoBio PowerSoil extraction kits under manufacturer's protocols (MoBio Inc). Approximately 40–200 mg of faecal material was used from each sample. Extracted DNA was quantified in a fluorometric assay using a Qubit fluorometer (Invitrogen Inc).

16S *rRNA* community profiles were characterized using Illumina MiSeq sequencing of the V4 region, and for a subset of samples using 454 pyrosequencing of the V1–V3 regions (sequencing both regions permitted comparison to a broader range of publicly available data sets). PCR amplification and Illumina sequencing of the 16S V4 region were performed at Argonne National Laboratories (Lemont, IL), following previously published protocols^33^,^34^. Briefly, 1 μl aliquots of extracted DNA were amplified in triplicate 25 μl PCR reactions, using barcoded universal fusion primers 515 forward (5′-GTGYCAGCMGCCGCGGTAA-3′) and 806 reverse (5′-GGACTACNVGGGTWTCTAAT-3′), at a 60 °C annealing temperature, for 35 cycles. Triplicate aliquots were then combined per sample, normalized to equimolar concentrations and pooled for sequencing on an Illumina MiSeq sequencer using paired-end, 250 bp reads. PCR amplification and 454 pyrosequencing of the V1–V3 regions were both performed at Research and Testing Laboratories (Lubbock, TX) using previously published protocols^35^. Briefly, singlicate amplifications were performed in 25-μl reactions, using universal fusion primers 27 forward (5′-AGAGTTTGATGMTGGCTCAG-3′) and 515 reverse (5′-TTACCGCGGCMGCSGGCAC-3′)^36^, an annealing temperature of 54 °C and 1 μl of template. Normalized equimolar concentrations of PCR product were then pooled and sequenced using 454 Titanium chemistry (Roche).

For a subset of samples, we also performed shotgun metagenomic sequencing. We prepared libraries for six cetacean specimens (JS1, JS2, F5, F8, F12 and F16) using the PrepX ILM automated DNA library prep kit (WaferGen Biosystems; Fremont, CA) and NextFLEX Illumina-compatible barcode adapters (BIOO Scientific; Austin, TX) according to the manufacturer's recommended protocol, with starting input DNA quantities ranging from 2.5 to 18 ng and size selection post-ligation via automated double-ended magnetic bead cleanup. Prior to library preparation, samples were sonically sheared in 50 μl of water using a Covaris S220 focused-ultrasonicator tuned to 400-bp fragment size (Covaris Inc). Size-selected libraries were then amplified for 14 cycles using NEBNext High-fidelity polymerase according to manufacturer's protocols (New England Biolabs Inc), except that amplifications took place in duplicate 25-μl reactions. Post-amplification, libraries were purified by hand using magnetic beads (AMPure, Agencourt Inc). Amplified libraries were then quantified via Bioanalyzer (High Sensitivity DNA assay, Agilent Technologies Inc) and qPCR (KAPA Library Quantification Kit; KAPA Biosystems Inc), pooled in equimolar concentrations and sequenced on an Illumina HiSeq 2,500 instrument using paired-end 150-bp chemistry.

To extend our metagenomic data set to a wider array of taxa, and to better match the longer read length available for previously published metagenomic data sets, we also constructed an additional 14 metagenomic libraries using Illumina MiSeq paired-end 250 bp chemistry. These libraries included seven of our terrestrial or aquatic samples (JS3, JS4, JS5, JS6, JS7, JS8 and JS19), five additional cetacean samples (JS10, JS13, JS17, F9 and F11) and two technical replicates of samples previously sequenced (F12 and F16). Prior to library preparation, samples were sonically sheared in 50 μl of water using a Covaris S220 focused-ultrasonicator tuned to 400-bp fragment size. All 14 of these libraries were prepared using the KAPA LTP library preparation kit for Illumina and NextFLEX Illumina-compatible barcode adapters according to the manufacturer's recommended protocol, with starting input DNA quantities ranging from 8.4 to 180.6 ng, and size selection post-ligation via double-ended magnetic bead cleanup. Size-selected libraries were amplified for either 8 cycles (JS3, JS4, JS10, JS19) or 10 (remaining samples) using KAPA HiFi DNA polymerase amplification kits according to manufacturer's protocols, except that amplifications took place in duplicate 25-μl reactions. Amplified libraries purified and quantified as described above, pooled in equimolar concentrations and sequenced on an Illumina MiSeq instrument using paired-end 250-bp chemistry.

### 16S community sequence analysis

To maximize our ability to place the whale microbiota in the broader context of mammalian gut community diversity, we focused our analysis on Illumina sequences from the V4 region of 16S, for which the greatest diversity of mammalian microbiota samples were available at the time of analysis. From the Earth Microbiome Project database, we downloaded Illumina V4 data sets for a variety of myrmecophagous mammals^26^ and for the subset of the mammals from the study by Muegge *et al*.^4^ that were resequenced at this region for the study by Delsuc *et al*.^26^. Because a number of the myrmecophagous mammalian samples from the study by Delsuc *et al*.^26^ were identified as being potentially contaminated by environmental bacteria, these samples were removed prior to further analysis. Since the sequences from the Earth Microbiome Project were limited to the first 100 bases of the forward read, we trimmed our own sequences to match. After demultiplexing in QIIME, we concatenated sequences for all three data sets and proceeded with analyses.

Sequences were *de novo* clustered at 97% sequence identity and chimeras removed using UPARSE, following the procedure recommended by Edgar^37^. Chimeras were identified using both *de novo* detection and using the Greengenes database (12_10 release) as a reference^38^. Following OTU picking, a QIIME-compatible OTU map was created using a custom python script. All subsequent analytical steps were performed in QIIME version 1.8.0 (ref. 39). Clustered, putatively nonchimeric sequences were assigned taxonomies using the RDP classifier^40^ trained on the aforementioned Greengenes database at an 80% confidence level. Representative sequences from each OTU were aligned to Greengenes and filtered of hypervariable positions using PyNast^41^, and a *de novo* phylogenetic tree was computed using FastTree^42^ within QIIME. For downstream analyses, OTU tables were rarified to 10,000 sequences, retaining the 4 samples (JS2, JS3, JS4 and JS12) with lower sequence counts. Unweighted UniFrac pairwise distance matrices were calculated in QIIME, and visualized using NMDS ordination with the vegan package in R 2.15 (http://www.cran.r-project.org/package=vegan). To enable comparison with a previously-published data set of wild seal microbiota^43^, we downloaded that data set from MG-RAST and repeated the above steps for 454 sequences from the V1–V3 regions of 16S.

Some studies have found the retention of DNA from food-derived bacteria in faecal samples^11^. To help ensure that the sequences derived from whale faeces were not in large part derived from their food organisms, we screened all samples analysed in this study against a library of 231 full-length bacterial 16S sequences derived from calanoid copepods in the North Atlantic^44^, the major food source of the right whales in our study. The representative sequence from each 97% OTU was searched against the copepod database using BLAST with an e-value cut-off of 10^−30^ and a per cent identity cut-off of 97%. Hits were inspected to ensure the returned alignments were at least 75 bp in length.

We used both OTU and taxonomy-based approaches to identify bacterial lineages that were enriched in whales relative to other mammals. For OTU-based approaches, we utilized Kruskal–Wallace and nonparametric pairwise T significance tests in QIIME to identify specific *de novo* OTUs that were distributed significantly differently among categories. For taxonomy-based approaches, we summarized the rarified OTU table by family-level taxonomy in QIIME, then imported relative abundances from this table into the Galaxy implementation of LEfSe^45^.

### Shotgun metagenomic sequence analysis

We used analysis of shotgun metagenomic sequences to explore differences in functional capacity between gut microbiomes of whales and other mammals. To maximize the breadth of possible comparisons, we combined our data with previously published mammalian microbiome data from the study by Muegge *et al*.^4^, available on the MG-RAST database^46^. To minimize the potential effect of differences in sequence processing between data sets, we ran our metagenomic sequences through the MG-RAST pipeline before analysis. After removing adapter contamination with CUTADAPT^47^, demultiplexed sequences were uploaded to MG-RAST using recommended settings for paired-end sequence data. After processing, filtered, dereplicated predicted open reading frames (files labelled *.299.* in the MG-RAST naming hierarchy) and translated amino acid sequences (labelled *.350.*) were downloaded for both of our data sets (HiSeq and long-read MiSeq), as well as the Muegge^4^ 454 data set.

We used the HMP Unified Metabolic Analysis Network programme^48^ to estimate normalized abundances of metabolic genes and pathways. For all samples, predicted amino acid sequences were compared against the KEGG database release 62 (ref. 49) using the Ublast implementation of Usearch version 7.0.1001 (ref. 50), utilizing an acceleration parameter of 0.5 and an e-value cut-off of 10^−5^. For the six more deeply sequenced HiSeq samples, a random subset of 105,191 sequences were tested (equal to the number of predicted coding sequences in the most deeply sequenced sample from the Muegge^4^ data set). Some samples, notably the toothed whales, showed signs of heavy contamination from host genomic sequences (Supplementary Fig S16). As a lack of available cetacean reference genomes precluded read mapping as a method for removing this contamination, we simply excluded reads for which the top Ublast hit was derived from a eukaryote.

Profiles of normalized, non-eukaryotic KEGG gene abundances were analysed using the Galaxy implementation of LEfSe^45^ and in R version 2.15 (http://www.r-project.org). KEGG genes and pathways significantly enriched in whales relative to other mammals were calculated in LEfSe. To visualize similarity among sample categories, we used redundancy analysis in the vegan statistical package in R (http://www.cran.r-project.org/package=vegan) to construct ordinations of KEGG genes and gene categories. Ordinations were calculated for the data set as a whole, as well as for subsets of genes and pathways defined by higher levels of organization in the KEGG gene ontology. For two sets of metabolic genes that have previously been identified as significant differentiators of herbivorous and carnivorous diets—namely, central pyruvate metabolism and central glutamate metabolism^4^,^11^—we manually curated lists of KEGG gene identifiers. We then used analysis of variance and Tukey's *post hoc* tests to determine significant differences between host categories.

### Analysis of carbohydrate-active enzymes

Predicted proteins were annotated following previously published methods^51^. First, unassembled metagenomic data sets were annotated against the CAZy database^52^ as follows. A local database of all predicted proteins corresponding to each family from the CAZy online database (http://www.cazy.org/, accessed 27 January 2014) was constructed and used to align predicted proteins using BLASTP (v 2.2.28+) (e-value cut-off of 1e−05). Predicted proteins were then annotated against the protein family (Pfam) database^53^ (ftp://ftp.ncbi.nih.gov/pub/mmdb/cdd/, accessed: 27 January 2014) using RPSBLAST (v 2.2.28+)^54^ (e-value cut-off of 1e−05). A CAZy-to-Pfam correlation list was compiled based on the secondary annotations provided through the CAZy online database. Only those proteins that had significant BLAST hits to a protein from our local CAZy database and its corresponding Pfam were retained and designated as a carbohydrate-associated enzyme. If no corresponding Pfam was identified, only CAZy hits with a bit score >60 were retained. Next, we generated a table with rows corresponding to samples and columns corresponding to each CAZy family. Because some proteins may encode for several CAZy families^52^, we allowed multiple CAZy annotations for individual proteins where appropriate. This table was quantile normalized using the default parameters in the metagenomeStats package in R version 3.0.2. For heatmap visualization and clustering, we applied Pearson's correlation coefficient index to the normalized data, clustered with average linkage UPGMA clustering and produced the figure using the gplots package in R. Significance tests were performed on the normalized data matrix using the Galaxy implementation of LEfSe, with a *P* value cut-off of 0.05 and a minimum effect size of 3.

## Additional information

**Accession codes:** Sequence data generated for this project have been deposited in the MG-RAST database under the project accession code mgp3854.

**How to cite this article:** Sanders, J. G. *et al*. Baleen whales host a unique gut microbiome with similarities to both carnivores and herbivores. *Nat. Commun.* 6:8285 doi: 10.1038/ncomms9285 (2015).

## Supplementary Material

Supplementary Information Supplementary Figures 1-9, Supplementary Table 1, Supplementary Discussion and Supplementary References

Supplementary Data 1 Output from LEfSe analysis of taxonomic categories as biomarkers of baleen whales, toothed whales, and terrestrial mammals.

Supplementary Data 2 Output from LEfSe analysis of taxonomic categories as biomarkers of baleen whales vs. right whales.

Supplementary Data 3 Results from group significance testing of individual OTUs in QIIME. Separate spreadsheet tabs include results for Kruskall-Wallace tests of differential distribution among dietary categories and among baleen whales / toothed whales / terrestrial mammals; and for non-parametric T-tests between terrestrial and marine mammals, baleen whales and dolphins, whales and terrestrial mammals, and whales and all other samples.

Supplementary Data 4 Summarized results of BLAST searches against the NCBI nt database for the ten most abundant OTUs in right whale, rorqual, and toothed whale libraries.

Supplementary Data 5 Output from LEfSe analysis of normalized CAZyme abundance as biomarkers of baleen whales vs. terrestrial mammals. For significant CAZyme biomarkers, the gene family description from dbCAN is included 2.
