# Supplementary material for: Baleen whales host a unique gut microbiome with similarities to both carnivores and herbivores
Source: Nat Commun. 2015 Sep 22;6:8285. doi: 10.1038/ncomms9285 (PMC4595633; doi:10.1038/ncomms9285)
Supplement: Supplementary Information — Supplementary Figures 1-9, Supplementary Table 1, Supplementary Discussion and Supplementary References [file ncomms9285-s1.pdf]

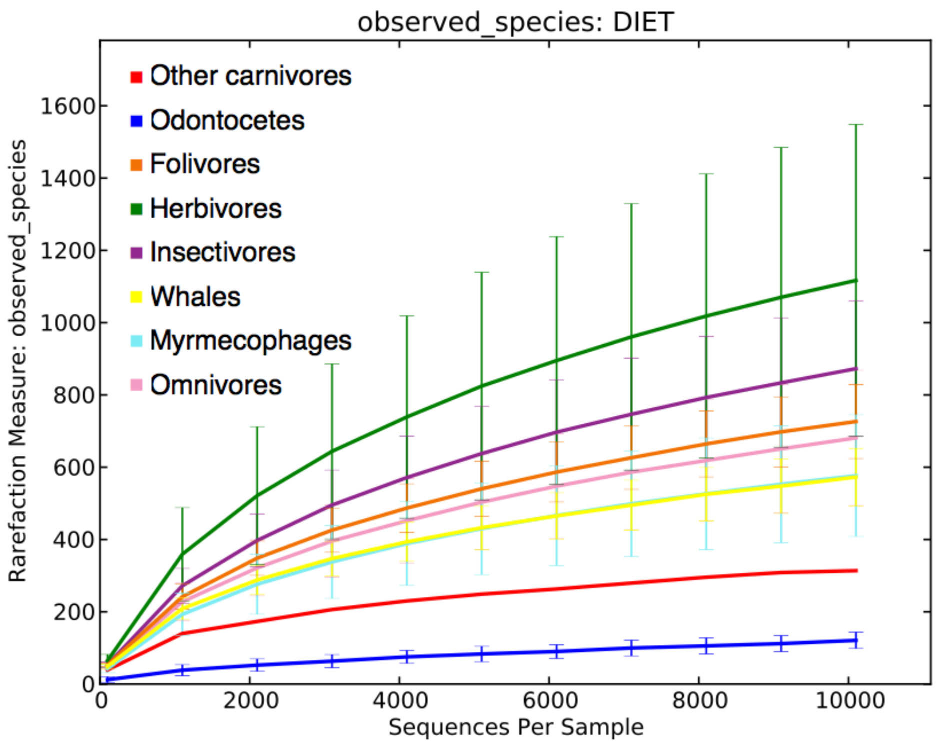

Supplementary Fig. 1: 16S rRNA rarefaction curves indicating mean alpha diversity (observed 97% OTUs) for different mammalian dietary categories, error bars indicating standard deviations. Odontocetes had the lowest observed diversity, followed by non-insectivorous carnivores, whales, and myrmecophages.

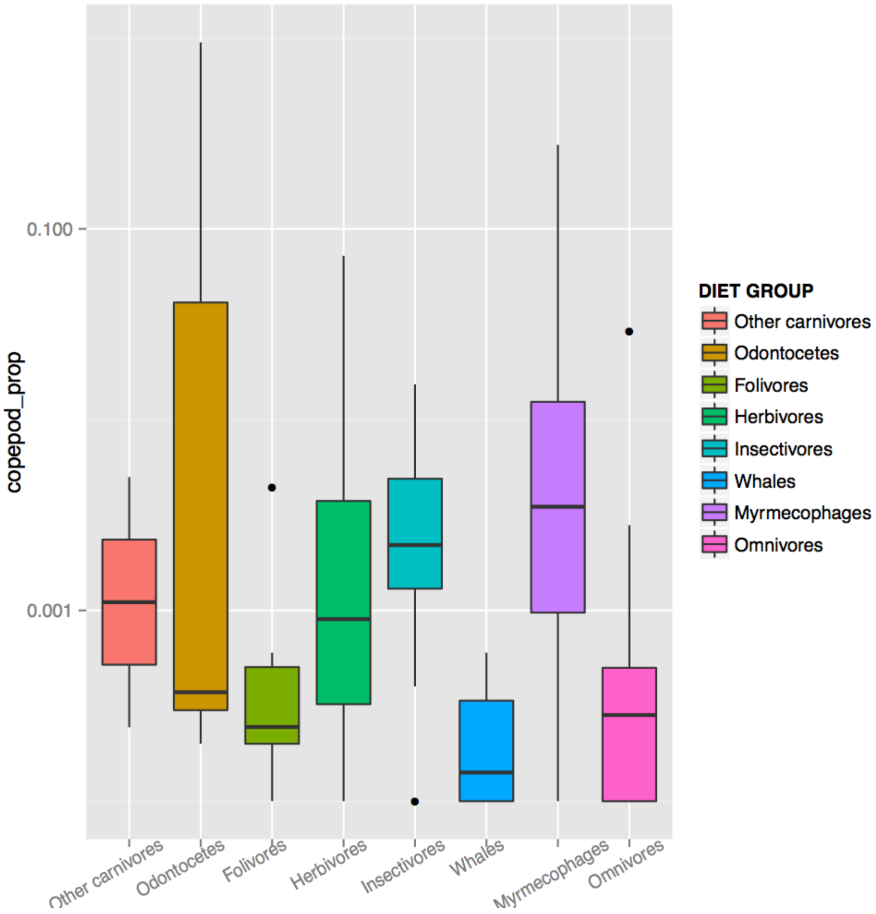

Supplementary Fig. 2: Whale microbiota are not enriched in copepod-associated sequences. Boxplot of proportion of sequence reads that BLAST to a database of bacterial 16S sequences associated with North Atlantic calanoid copepods, the preferred food of the right whales in this study. Y axis is log transformed.

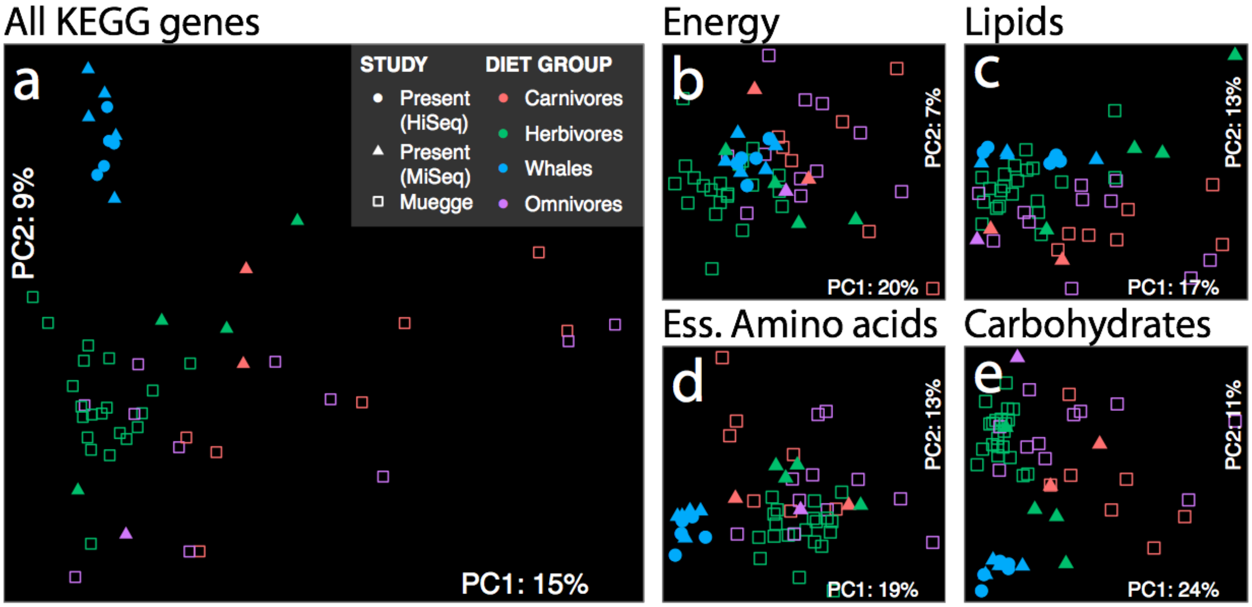

Supplementary Fig. 3: Unconstrained RDA ordinations of KEGG gene abundances in microbiomes of whales, other carnivores, herbivores, and omnivores; identical to Figure 2, except calculated using individual abundances rather than pathway abundances. Note that in (d), whales group separately from terrestrial mammals, whereas when considering whole pathway abundances, they group together with the terrestrial carnivores.

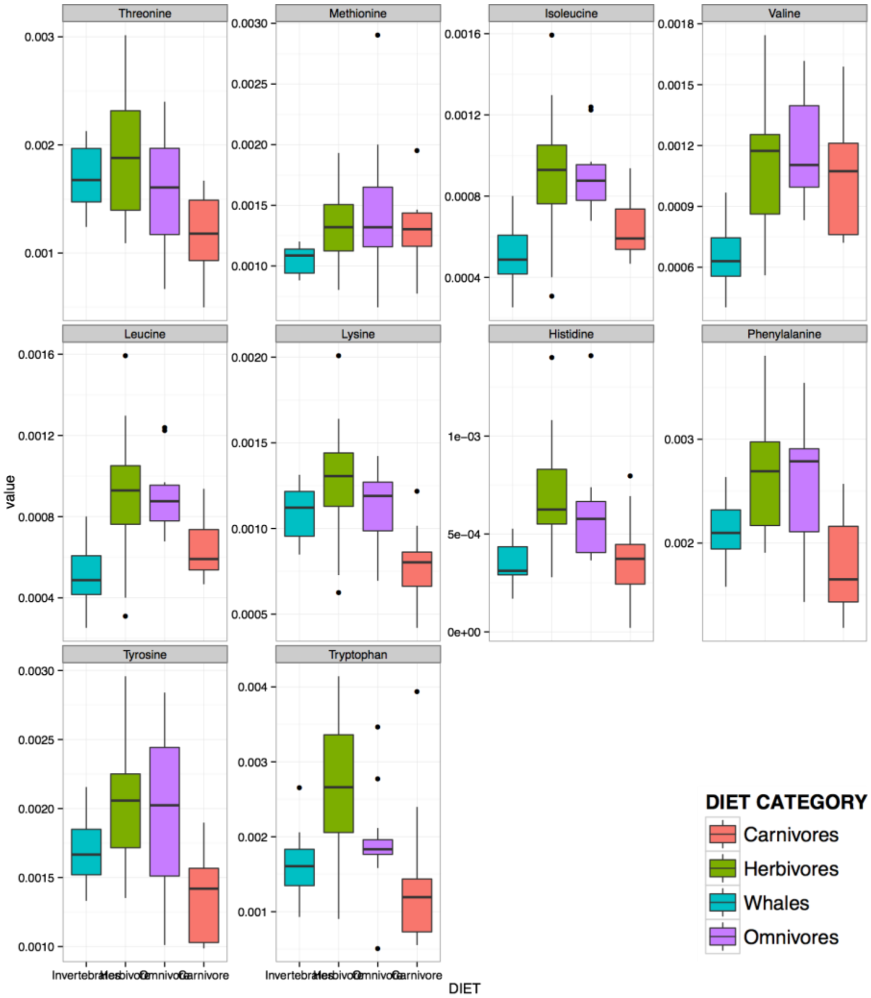

Supplementary Fig. 4: Normalized abundances for genes catalyzing the final step of biosynthesis for the nine essential amino acids. According to Mann-Whitney post-hoc pairwise tests, whale microbiomes are significantly depleted relative to herbivore microbiomes for all but Threonine, Lysine, and Tyrosine; they are significantly enriched relative to terrestrial carnivores only for Threonine and Lysine.

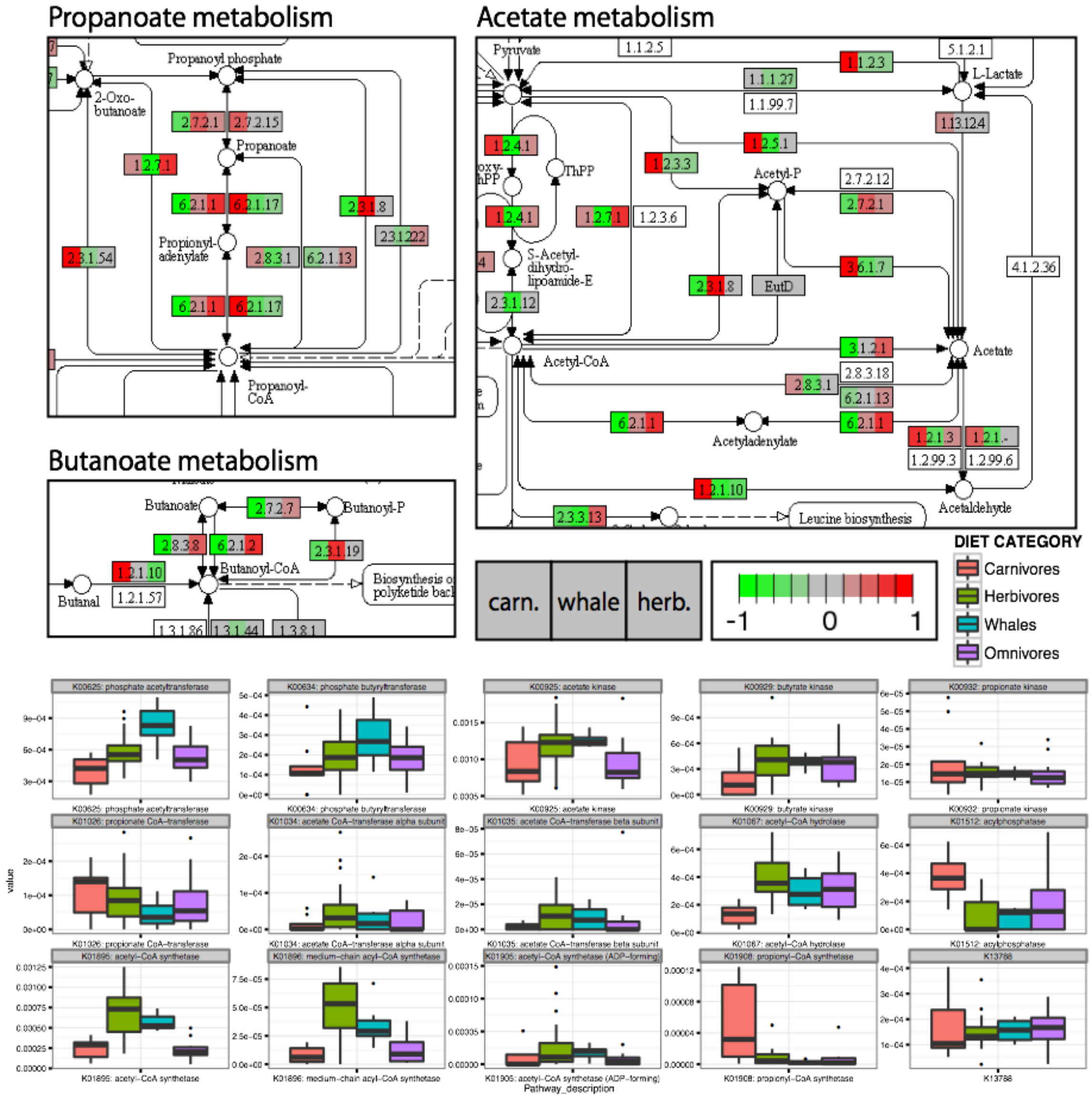

Supplementary Fig. 5: Enrichment of SCFA-associated genes. KEGG pathway vignettes and normalized gene abundances indicating the relative enrichment of genes involved in the processing of the Short Chain Fatty Acids (SCFAs) acetate, propanoate, and butanoate. Genes performing each catalytic function (represented by E.C. numbers) are indicated for carnivores, baleen whales, and herbivores are represented for each E.C. number in that order. Gene abundances are normalized, mean-centered values, and averaged by dietary category; genes normalized including omnivore samples, but only carnivores, whales, and herbivores are displayed. Red boxes indicate that the average normalized abundance for that dietary category is greater than the global average for that gene; green, that it is less than average.

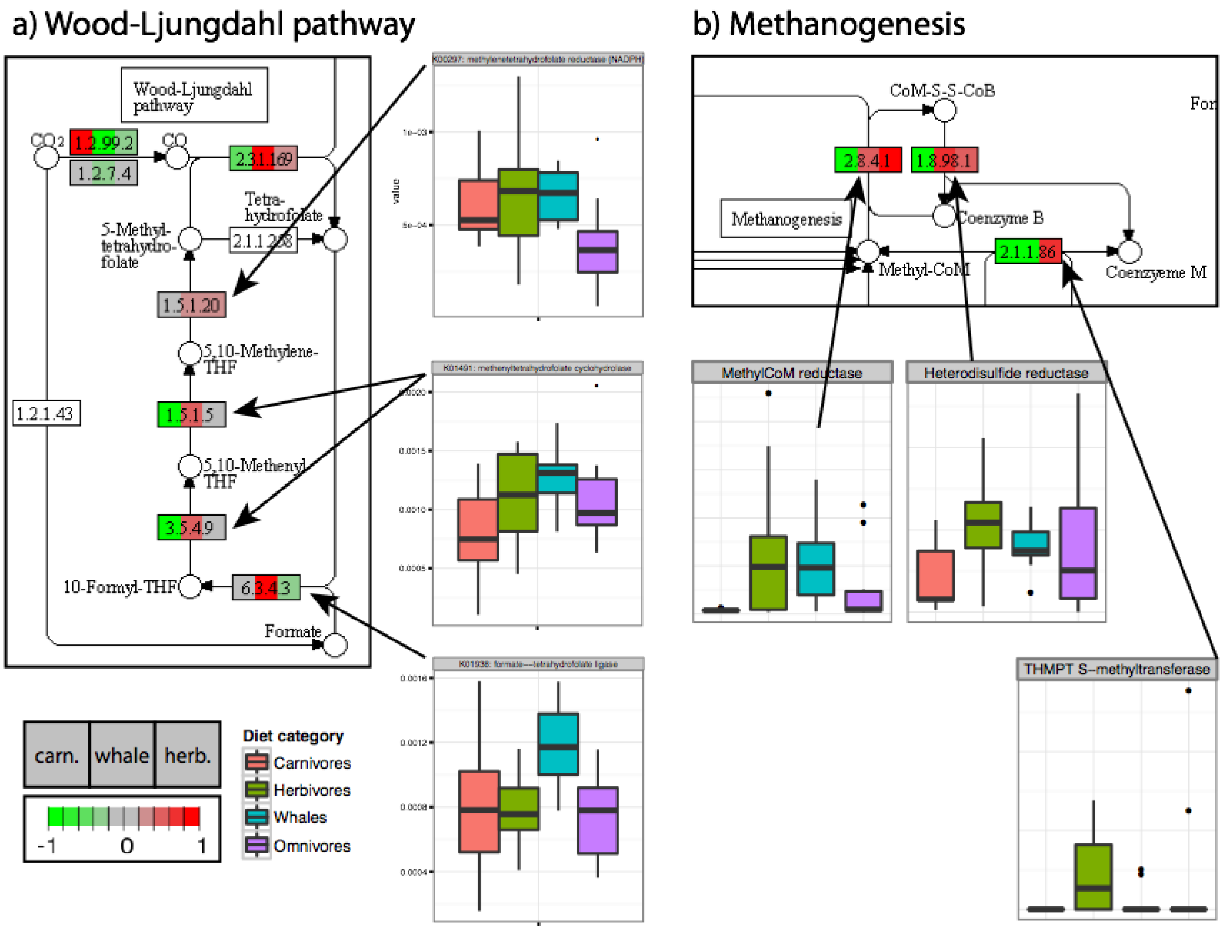

Supplementary Fig. 6: Genes associated with fermentation. a) Relative abundance of key genes in the Wood-Ljungdahl pathway. KEGG pathway vignette is colored as in Supplementary Figure 5. b) Relative abundance of key genes in methanogenesis. Box plots include the summed relative abundance of all annotated subunits for each enzyme.

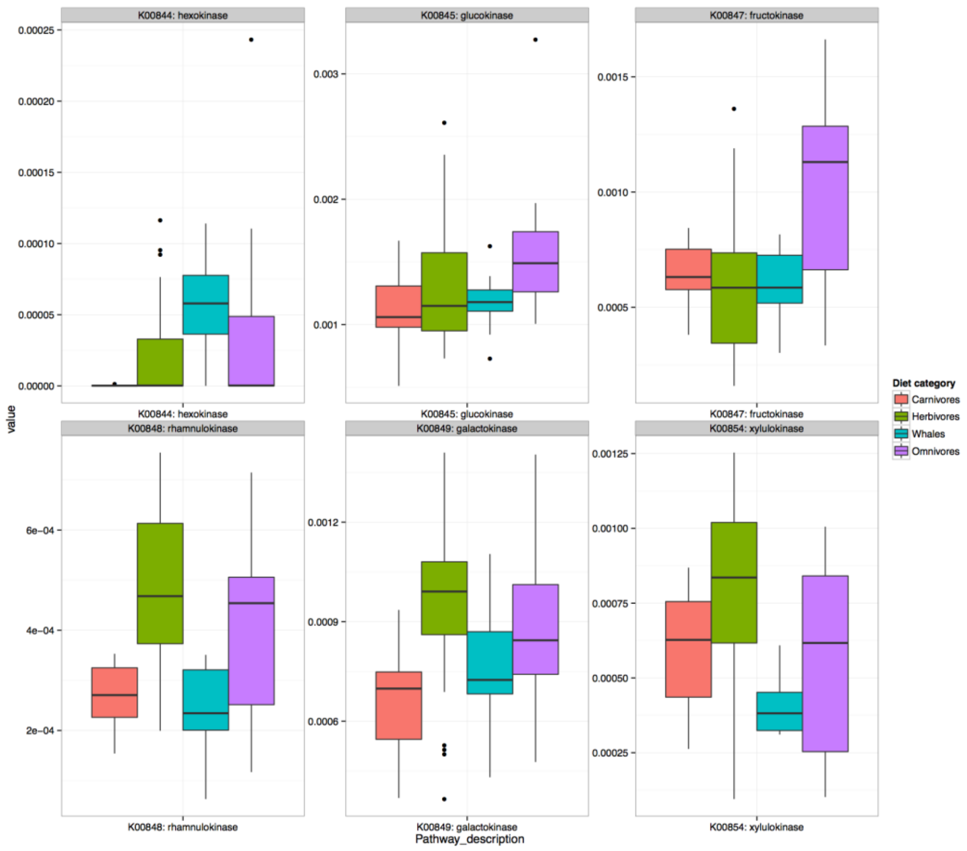

Supplementary Fig. 7: Relative abundances of monosaccharide kinases in mammal microbiomes. Like terrestrial carnivores, whales were relatively depleted in kinases for sugars commonly found in hemicellulose.

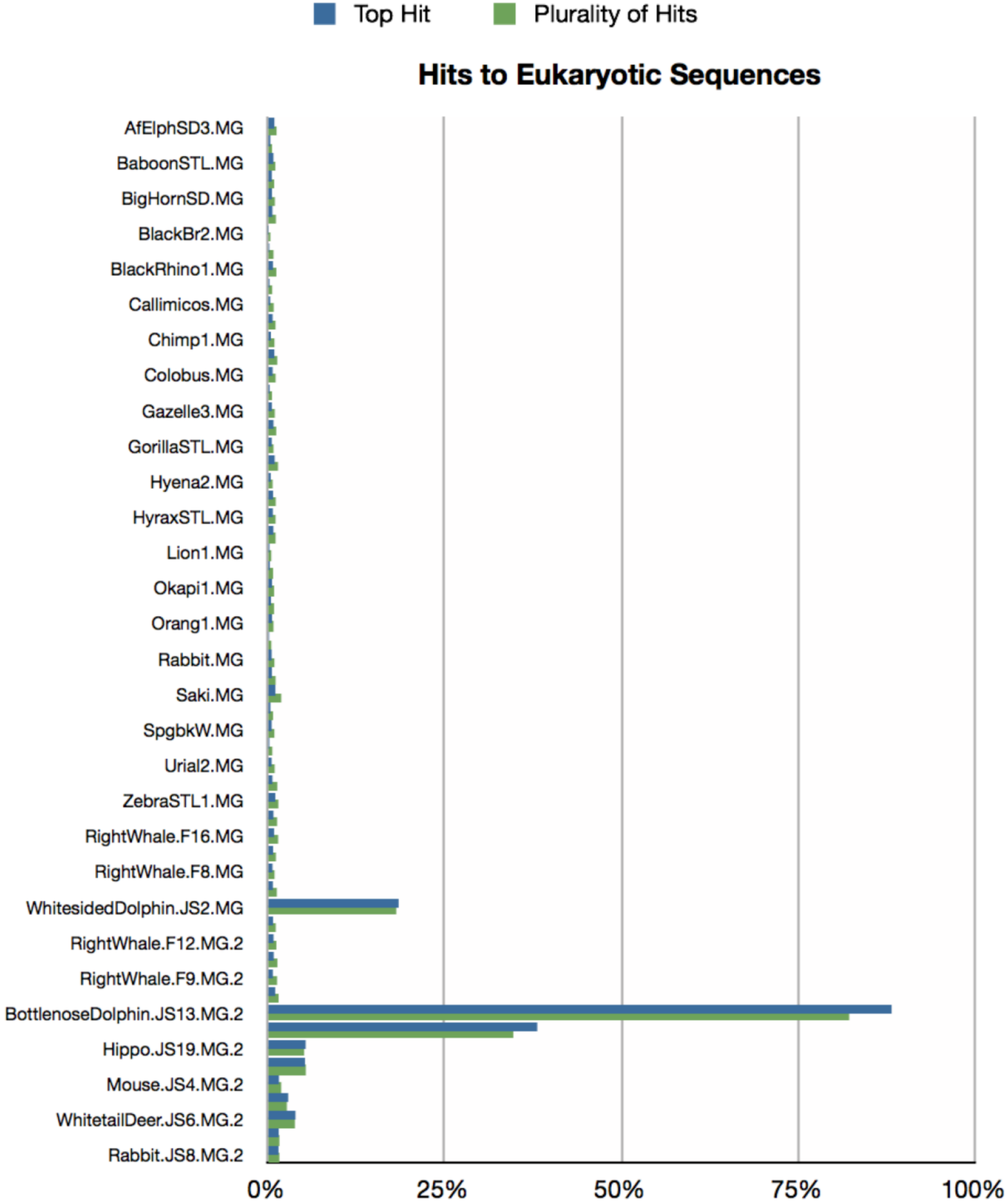

Supplementary Fig. 8: Likely host contamination as a proportion of overall reads for each dataset. Blue bar in each pair indicates proportion of reads that matched to a eukaryotic sequences as top hit; green bar indicates proportion of reads for which a plurality of top hits were from eukaryote.

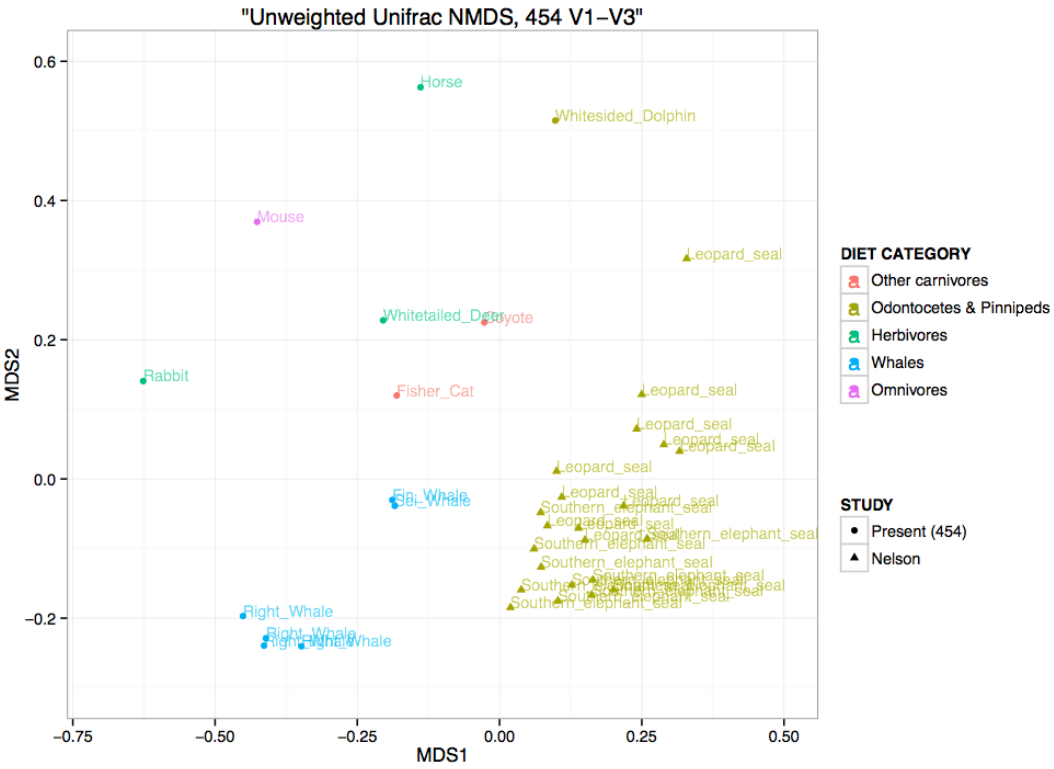

Supplementary Fig. 9: NMDS ordination of unweighted UniFrac distances for pinniped samples from Nelson *et al* (Supplementary Ref 1) and the present study. Distances based on 97% OTUs clustered from 454 sequencing of the V1-V3 region of the bacterial 16S rRNA gene.

Host ID	Host Species	Collection Date	Collection Location[1]	454 16S (V1-V3)[2]	Illm 16S (V4)[3]	Meta[4]
JS1	Sei Whale (*Balaenoptera borealis*)	18 Sep 2011	Grand Manan, NB, Canada	Yes	Yes	
JS2	Atlantic Whitesided Dolphin (*Lagenorhynchus acutus*)	23 Aug 2011	Grand Manan, NB, Canada	Yes	Yes	
JS3	Coyote (*Canis latrans*)	15 Nov 2012	Concord, MA	Yes	Yes	Yes
JS4	Mouse (*Mus musculus*)	14 Dec 2012	Harvard University, MA	Yes	Yes	Yes
JS5	Horse (*Equus ferus caballus*)	9 Dec 2012	Concord, MA	Yes	Yes	Yes
JS6	White-tailed Deer (*Odocoileus virginianus*)	25 Nov 2012	Concord, MA	Yes	Yes	Yes
JS7	Fisher (*Martes pennanti*)	22 Nov 2012	Stow, MA	Yes	Yes	Yes
JS8	Rabbit (*Oryctolagus cuniculus*)	13 Dec 2012	Cambridge, MA	Yes	Yes	Yes
JS9	Humback Whale (*Megaptera novaeangliae*)	12 Oct 2008	Seymour Canal, AK		Yes	
JS10	Humback Whale (*Megaptera novaeangliae*)	22 Jun 2011	Sitka Sound, AK		Yes	Yes
JS11	Humback Whale (*Megaptera novaeangliae*)	15 Nov 2009	Seymour Canal, AK		Yes	
JS12	Humback Whale (*Megaptera novaeangliae*)	15 Nov 2009	Seymour Canal, AK		Yes	
JS13	Bottlenose Dolphin (*Tursiops truncatus)*	22 Apr 2011	Long Marine Laboratory, CA		Yes	Yes
JS14	Bottlenose Dolphin (*Tursiops truncatus)*	22 Apr 2011	Long Marine Laboratory, CA		Yes	
JS16	Beluga (*Delphinapterus leucas*)	28 May 2011	Mystic Aquarium, CT		Yes	
JS17	Beluga (*Delphinapterus leucas*)	28 May 2011	Mystic Aquarium, CT		Yes	Yes
JS18	Hippopotamus (*Hippopotamus amphibius*)	22 Aug 2012	Narok County, Kenya		Yes	
JS19	Hippopotamus (*Hippopotamus amphibius*)	22 Aug 2012	Narok County, Kenya		Yes	Yes
JS20	Hippopotamus (*Hippopotamus amphibius*)	21 Aug 2012	Narok County, Kenya		Yes	
JS21	Hippopotamus (*Hippopotamus amphibius*)	21 Aug 2012	Narok County, Kenya		Yes	
F2	Right Whale (*Eubalaena glacialis*)	13 Aug 2011	Grand Manan, NB, Canada		Yes	
F5	Right Whale (*Eubalaena glacialis*)	13 Aug 2011	Grand Manan, NB, Canada	Yes	Yes	
F8	Right Whale (*Eubalaena glacialis*)	23 Aug 2011	Grand Manan, NB, Canada	Yes	Yes	
F9	Right Whale (*Eubalaena glacialis*)	24 Aug 2011	Grand Manan, NB, Canada		Yes	Yes
F11	Right Whale (*Eubalaena glacialis*)	24 Aug 2011	Grand Manan, NB, Canada		Yes	Yes
F12	Right Whale (*Eubalaena glacialis*)	24 Aug 2011	Grand Manan, NB, Canada	Yes	Yes	Yes
F16	Right Whale (*Eubalaena glacialis*)	2 Sep 2011	Grand Manan, NB, Canada	Yes	Yes	Yes

Supplementary Table 1: Detailed collection information for samples in this study.
[1]All collection locations in USA unless specified. NB: New Brunswick; MA: Massachusetts; AK: Alaska; CA: California; CT: Connecticut. [2]454 sequencing of PCR amplicons of the V1-V3 regions of the 16S rRNA gene performed for this sample. [3]Illumina sequencing of PCR amplicons of the V4 region of the 16S rRNA gene performed for this sample. [4]Illumina shotgun metagenomic sequencing performed for this sample.

Supplementary Discussion

Toothed whales

In contrast to baleen whales, the toothed whale samples in our dataset were highly variable in taxonomic composition, often dominated by one or two bacterial lineages typically found at lower abundance in other mammalian guts (Fig 1a). Some of these dominant OTUs were perfect matches to sequences previously recovered from marine environmental samples, including the Gammaproteobacteria *Pseudoalteromanas* and *Photobacterium*, suggesting the potential for an environmental origin. Some of these sequences also matched a library of bacterial 16S sequences associated with marine copepods, further suggesting the potential impact of environmental bacteria (Supplementary Fig. 2). However, three other dominant OTUs were 99-100% matches to sequences previously recovered from other marine mammals, including *Mycoplasma* (Tenericutes), *Mycobacterium* (Actinobacteria), and *Cetobacterium* (Fusobacteria). In aggregate, the taxonomic composition we observed in toothed whales showed a similar trend to what has previously been reported for piscivorous pinnipeds [1,2], with an enrichment in Proteobacteria and Fusobacteria relative to terrestrial mammals.

The three toothed whale metagenomes also showed notable differences to the overall pattern of similarity with 16S data, with these samples having dramatically different profiles of predicted functional genes. Taxonomic profiling suggests an extremely high degree of host genomic contamination for these samples: between 20 and 90% of reads matched most closely to eukaryotic sequences in the KEGG

database (Supplementary Fig. 8). Manual inspection of these reads reveals that most match closely to the low-coverage genomic sequence available for the dolphin *Trusiops truncatus* (Ensembl accession *turTru1*) or to *Bos taurus*, suggesting that the sequenced DNA was derived from the host animal rather than undigested food or laboratory contamination. The source of this host DNA is unknown, but may arise from sloughing intestinal epithelia in a midgut that is much longer than in most other mammals [3]. Together with the high taxonomic variability observed in 16S data from these samples, the high proportion of host sequence suggests that these samples may not be directly comparable to other samples; we have consequently excluded them from further analysis.

Difference among whales

Comparison of whale species within our sample set also showed moderate differences in microbial composition, potentially reflecting differences between diets. Our Illumina 16S data consisted of seven right whales, which feed primarily upon crustaceans such as calanoid copepods, and five other baleen whales (humpback and sei whales), which consume both vertebrate and invertebrate prey. Compared with right whales, other baleen whales appeared to retain more of the bacterial taxa that were also more abundant in terrestrial mammals, such as Bacilli, *Blautia*, *Coprococcus*, and *Coprobacillus,* than did right right whales (Supplementary Data 2). Diets consisting entirely of invertebrates would be likely to contain higher proportions of fermentable animal polysaccharides (including chitin). Consistent with this, the clade 5 Verrucomicrobia, which were more enriched in baleen whales and terrestrial herbivores

than in other mammals, were also significantly more abundant in right whales than in other baleen whales. These overall differences in community composition were especially apparent in an initial 454 dataset of the V1-V3 regions of 16S, with baleen whales grouping separately from both terrestrial samples and from Antarctic seals (Supplementary Fig. 9).

Supplementary References

1. Nelson, T. M., Rogers, T. L., Carlini, A. R. & Brown, M. V. Diet and phylogeny shape the gut microbiota of Antarctic seals: a comparison of wild and captive animals. *Environmental Microbiology* **15,** 1132–1145 (2012).
2. Nelson, T. M., Rogers, T. L. & Brown, M. V. The gut bacterial community of mammals from marine and terrestrial habitats. *PLoS ONE* **8,** e83655 (2013).
3. Williams, T. M., Haun, J., Davis, R. W., Fuiman, L. A. & Kohin, S. A killer appetite: metabolic consequences of carnivory in marine mammals. *Comp Biochem Phys A* **129,** 785–796 (2001).
